# Supplementary material for: Urinary Continence Recovery after Robotic Radical Prostatectomy without Anterior or Posterior Reconstruction: Experience from a Tertiary Referral Center
Source: J Clin Med. 2023 Feb 8;12(4):1358. doi: 10.3390/jcm12041358 (PMC9962972; doi:10.3390/jcm12041358)
Supplement: Supplementary file 1 [file jcm-12-01358-s001.zip › Supplementary Table S1.pdf]

**Table S1. Patient's and tumour's characteristics for those receiving MRI**

| **Variables** | **n=518** |
|---|---|
| *Patient's characteristics* | |
| Age at diagnosis, yrs, median (IQR) | 67 (62 - 71) |
| BMI at surgery, kg/m2, median (IQR) | 26 (24 - 28,4) |
| Charlson Comorbidity index (CCI) | 1 (0-2) |
| Diabetes mellitus, n (%) | 40 (7,7) |
| Anticoagulant/antiaggregant, n (%) | 121 (23,4) |
| Family history of prostate cancer, n (%) | 60 (11,6) |
| Preoperative urinary incontinence (UI)*, n (%) | 6 (1,2) |
| Preoperative ED, n (%) | 91 (17,6) |
| Positive DRE, n (%) | 247 (47,7) |
| PSA value at diagnosis, ng/mL, Median (IQR) | 6.3 (4.3 - 9.1) |
| Prostate Volume, cc, Median ° (IQR) | 45 (34.0 – 58.0) |
| *Characteristics at biopsy* | |
| Type of biopsy, n (%)<br>- Cognitive Biopsy<br>- MRI-targeted biopsy | <br>170 (32,8)<br>348 (67,2) |
| Highest ISUP Grade Group at MRI targeted – biopsy cores, n (%)<br>- Patients with no positive random – biopsy cores<br>- Grade Group 1<br>- Grade Group 2<br>- Grade Group 3<br>- Grade Group 4<br>- Grade Group 5 | <br>185 (35,7)<br>39 (7,5)<br>109 (21,0)<br>108 (20,8)<br>37 (7,1)<br>40 (7,7 |
| Extra Capsular Invasion at biopsy, n (%) | 5 (1,0) |
| Seminal Vesicles Invasion at biopsy, n (%) | 0 (0) |
| Perineural Invasion at biopsy (PNI), n (%) | 116 (22,4) |
| *Characteristics at imaging* | |
| Extra Capsular Extension at imaging, n (%) | 30 (5,8) |
| Seminal Vesicles Invasion at imaging, n (%) | 11 (2,1) |
| cT, n(%)<br>- T1<br>- T2<br>- T3-4 | 213 (41,1)<br>282 (54,4)<br>23 (4,4) |
| cN+, n (%) | 27 (5,2) |
| Localized disease § | 475 (91,7) |
| Novel risk categories , n(%)<br>- Group 1-low-risk<br>- Group 2- intermediate risk<br>- Group 3- high risk<br>- Group 4- veriy high risk | <br>43 (8,3)<br>312 (60,2)<br>152 (29,3)<br>11 (2,1) |

*UI defined according to EPIC criteria as the need for of more than one Pad per day;° Prostate volume was assessed at sovrapubic ultrasound and/or MRI, if available; Ç The grade group was evaluated only on patients with random samples at biopsy (n = 925). If case of multiple Gleason scores in the same patient, the highest was considered.; § Localized disease was defined as the presence of cT1-2, N0 M0 prostatic cancer, according to EAU Guidelines

BMI: body mass index; ED: erectile dysfunction; MRI: magnetic resonance imaging; TURP: transurethral resection of prostate
