# Supplementary material for: Urinary Continence Recovery after Robotic Radical Prostatectomy without Anterior or Posterior Reconstruction: Experience from a Tertiary Referral Center
Source: J Clin Med. 2023 Feb 8;12(4):1358. doi: 10.3390/jcm12041358 (PMC9962972; doi:10.3390/jcm12041358)
Supplement: Supplementary file 1 [file jcm-12-01358-s001.zip › Supplementary Table S2.pdf]

Table S2. Intra and histopatological features.

**Variables**	**n=518**
Surgical Features	
Nerve sparing RALP, n (%)	304 (58,6,8)
Monolateral Nerve sparing RALP n (%)	165 (31,8)
Bilateral Nerve sparing RALP n (%)	139 (26,8)
Pelvic lymph node dissection during (LND) RALP, n (%)	237 (45,5)
Histopatologic features	
- Definitive histology findings, n (%)	
- Acinar adenocarcinoma	486 (93,8)
- Intraductal carcinoma	4 (0,8)
- Mixed	9 (1,7)
- Others (sarcomatoid, squamous and adenosquamous)	19 (3,7)
Clinically significant positive surgical margin (PSM) , more than 2mm, n (%)	40 (7,7)
Highest ISUP Grade Group Post-RP histopathologic assessment, n (%)	
- Grade Group 1	39 (7,6)
- Grade Group 2	256 (49,8)
- Grade Group 3	83 (16,1)
- Grade Group 4	82 (16,0)
- Grade Group 5	54 (10,5)
Gleason Score (GS) at definitive Post-RP histopathologic assessment , n (%)	
- GS 6 (3+3)	39 (7,6)
- GS 7 (3+4 and 4+3)	373 (72,6)
- GS 8 (4+4 and 5+3 and 3+5)	60 (11,7)
- GS 9 (4+5 and 5+4)	42 (8,2)
Perineural Invasion (IPN) at definitive Post-RP histopathologic assessment, n (%)	234 (45,2)
pT, n (%)	
- T2	214 (41,3)
- T3	302 (58,3)
- T4	2 (0,4)
pN, n (%)	
- Nx	281 (54,2)
- N0	189 (36,5)
- N1	48 (9,3)
pM, n (%)	
- Mx	490 (94,6)
- M0	26 (5,0)
- M1 (extraregional LND)	2 (0,4)
