# Supplementary material for: Urinary Continence Recovery after Robotic Radical Prostatectomy without Anterior or Posterior Reconstruction: Experience from a Tertiary Referral Center
Source: J Clin Med. 2023 Feb 8;12(4):1358. doi: 10.3390/jcm12041358 (PMC9962972; doi:10.3390/jcm12041358)
Supplement: Supplementary file 1 [file jcm-12-01358-s001.zip › Supplementary Table S3.pdf]

Supplementary Table S3. Post-operative features and follow-up of patients with MRI

**Variables**	**n=518**
Follow-up	
Duration of follow-up, months, Median (IQR)	14 (8-19)
Biochemical recurrence (BCR)at last follow-up	18 (3,5)
Of patients with BCR:<br>- adiuvant radiotherapy (in 4 cases concomitant ormonotherapy)<br>- salvage radiotherapy<br>- systemic ormonotherapy	 12 (42)<br>2 (12)<br>4 (14)
Early (30-days) continence rate, n (%)<br>- Fully continent, No pad/die<br>- 1 or more pads/die	 426 (82,3)<br>92 (17,7)
Continence at last follow-up , n (%)<br>- Fully continent, No pad/die<br>- 1 or more pads/die	 481 (93,05)<br>37 (6,95)
Clavien Dindo (CD) > 2 within 30 days), n(%)<br>- CD IIIa<br>*(6 cases requiring percutaneous drainage for symptomatic lymphocele, 5 cases 3 requiring endoscopic catheter positioning for mdc spread)*<br>- CD IIIb<br>*(4 endoscopic urethrotomy for ureteral stricture)*<br>- CD IV<br>*(1 case of post-operative cerebrovascular ischemia requiring neurovascular thrombolysis)*	 9 (2,1)<br><br><br>4 (0,7)<br><br>1 (0,1)
Clavien Dindo (CD) > 2 after 30 days), n (%)<br>- CD IIIa<br>*(7 cases requiring percutaneous drainage for symptomatic lymphocele, 4 percutaneous drainage for abdominal abscessus,*<br>- CD IIIb<br>*(8 endoscopic urethrotomy for endoscopic stricture,*<br>- CD IV<br>*(1 case of acute abdomen for bowel strangulation)*	 11 (2,1)<br><br>8 (1,5)<br><br>1 (0,1)
